# Comprehensive behavioral phenotyping of calpastatin-knockout mice

**DOI:** 10.1186/1756-6606-1-7

**Published:** 2008-09-15

**Authors:** Ryuichi Nakajima, Keizo Takao, Shu-Ming Huang, Jiro Takano, Nobuhisa Iwata, Tsuyoshi Miyakawa, Takaomi C Saido

**Affiliations:** 1 Laboratory for Proteolytic Neuroscience, RIKEN Brain Science Institute, Saitama, Japan; 2 Genetic Engineering and Functional Genomics Group, Frontier Technology Center, Graduate School of Medicine, Kyoto University, Kyoto, Japan; 3 Division of Systems Medicine, Institute for Comprehensive Medical Science, Fujita Health University, Toyoake, Japan; 4 JST, CREST, Hon-chou, Kawaguchi, Saitama, Japan; 5 Current Address: Department of Physiology & Biophysics, Dalhousie University Faculty of Medicine, Halifax, Nova Scotia, Canada; 6 Current Address: Department of Neuroscience, Institute for Chinese Medicine, Heilongjiang University of Chinese Medicine, Harbin, PR China

## Abstract

**Background:**

Calpastatin is an endogenous inhibitor of calpain, intracellular calcium-activated protease. It has been suggested to be involved in molecular mechanisms of long-term plasticity and excitotoxic pathways. However, functions of calpastatin in vivo are still largely unknown. To examine the physiological roles of calpastatin, we subjected calpastatin-knockout mice to a comprehensive behavioral test battery.

**Results:**

Calpastatin-knockout mice showed decreased locomotor activity under stressful environments, and decreased acoustic startle response, but we observed no significant change in hippocampus-dependent memory function.

**Conclusion:**

These results suggest that calpastatin is likely to be more closely associated with affective rather than cognitive aspects of brain function.

## Background

Calpastatin (CS) is the endogenous inhibitor of intracellular cysteine protease calpain. CS inhibits the Ca^2+^-activated form of calpain. In other words, calpain is bidirectionally regulated by Ca^2+ ^and CS, and this is called the "calpain-CS system". CS inhibits two forms of calpain: μ-calpain (calpain I) and m-calpain (calpain II), which are activated by micromolar and millimolar Ca^2+ ^in vitro, respectively [1].

The physiological roles of the calpain-CS system have not yet been well understood, though limited proteolysis by calpain is known to modify the functions of various substrates. Calpains are widely distributed in mammalian organs [2], and some important functions are already well known. For instance, the cyclin-dependent kinase 5 (Cdk5) activator, p35, is cleaved to p25 by calpain [3,4], and the generated p25 hyperactivates Cdk5, possibly leading to neurodegeneration. Another calpain-mediated neuronal death pathway involves the cleavage of Bid to generate tBid, resulting in DNA fragmentation [5]. The levels of CS in most organs of normal animals are sufficient to inhibit calpain [2], so CS can inhibit these calpain cascades.

The calpain-CS system is hypothesized to be involved in molecular processes of long-term potentiation (LTP) [6,7], which is considered to contribute to the synaptic changes associated with learning and memory [8-11]. One of the major calpain substrates in neurons is fodrin (spectrin), a cytoskeltal molecule that contributes to the post-synaptic structure, and this degradation of fodrin is inhibited by CS. Therefore, it is possible that the calpain-CS system contributes to the learning and memory processes, and there are several experiments that are related to the contributions of calpain-CS system on memory [12,13]. However, the calpain-CS system's involvement in learning and memory processes remains controversial.

To investigate the physiological roles of CS, we have generated CS knockout (KO) mice. In a previous study, CS-KO mice showed increased spectrin proteolysis following kainate administration, which suggested increased activity of calpain in such pathological conditions [5]. We also found increased LTP in CS-KO mice in both the hippocampal CA1 and dentate gyrus regions (Huang and Saido, unpublished data), even though no significant difference in LTP was detected in μ-calpain KO mice [12]. However, Grammer et al. also found a decreased paired pulse ratio in μ-calpain KO mice, suggesting a presynaptic modulatory role of μ-calpain [12]. In this report, we have subjected CS-KO mice to a systematic and well-defined comprehensive behavioral test battery [14-16], to clarify the physiological roles of CS in behavior.

## Results

### Physical features

Home cage behaviors and general health conditions of both the genotype groups, WT (wild-type) and CS-KO, appeared normal. Body weight and body temperature were not significantly different between the genotypes (F_1,36 _= 2.75, *P *= 0.106 for body weight, F_1,36 _= 0.320, *P *= 0.575 for body temperature). The appearance of fur and whiskers were not significantly different between the genotypes (Table 1).

**Table 1:** General characteristics of CS-KO mice.

	**WT**	**CS-KO**
***Number of animals***	20	18
***Physical characteristics***		
- Weight (g)	26.2 (± 0.33)	27.1 (± 0.40)
- Body temperature (°C)	36.9 (± 0.20)	37.1 (± 0.18)
- Whiskers (% with)	100	89
- Fur (% with normal fur)	100	100
***Sensory and motor reflexes***		
- Ear twitch (% with normal response)	100	83
- Key jangling (% with normal response)	95	89
- Whisker twitch	100	94
- Righting reflex	100	100
***Pain test***		
- Hot plate (latency; seconds)	6.75 (± 0.349)	6.05 (± 0.364)
***Motor tests***		
- Wire hang (% stayed up to 60 s)	95	94
- Grip strength (N)	0.893 (± 0.03)	0.825 (± 0.03)

No significant differences between genotypes were detected in physical characteristics (weight, body temperature, whiskers and fur), sensory and motor reflex (ear twitch response, key-jangling response and righting reflex), hot plate test (latency to the first paw response), and muscular abilities (number of animals that stayed up to 60 sec in wire hang test and grip strength).

### Neurological reflexes

Neurological reflexes were essentially normal in the CS-KO mice as compared with WT mice. Key jangling, whisker twitch response to a whisker touch from behind, and righting reflex were similar across genotypes (Table 1). Ear twitch responses tend to be decreased in CS-KO mice, but narrowly failed to achieve conventional measures of significance (*P *= 0.0594, *Student's *t-test).

### Pain sensation and motor abilities

In the hot plate test, latency to the first paw response was not affected by the lack of calpastatin (F_1,36 _= 1.93, *P *= 0.174). Muscular abilities appeared normal in terms of the wire hanging test across genotypes (F_1,36 _= 0.269, *P *= 0.607) and the grip strength test (F_1,36 _= 2.46, *P *= 0.126). (Table 1)

### Acoustic startle response and prepulse inhibition (sensorimotor gating)

**Figure 1:**
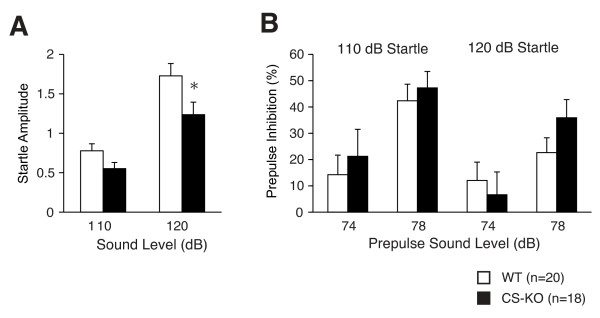
**Decreased acoustic startle response of CS-KO mice**. CS-KO mice displayed a significantly lower acoustic startle response (analyzed by repeated-measures ANOVA). Startle response caused by the 120 dB white noise was significantly lower in CS-KO mice (analyzed by ANOVA in each individual sound level experiments) (A). Prepulse inhibition was not significantly different across genotypes (B). (*) Significantly different in genotype effect, *P *< 0.05. Data are expressed as mean ± standard error of the mean (S.E.M.).

CS-KO mice displayed a significantly lower acoustic startle response than WT mice (repeated measures ANOVA, F_1,36 _= 4.98, *P *= 0.032; Figure 1A). Analysis of variance (ANOVA) in each individual sound level experiment detected a significant difference only in 120 dB session (in the 110 dB session, F_1,36 _= 3.57, *P *= 0.0670; in the 120 dB session, F_1,36 _= 4.87, *P *= 0.0338). Prepulse inhibition was not significantly different between genotypes (F_1,36 _= 0.419, *P *= 0.522 for 110 dB startle and F_1,36 _= 0.180, *P *= 0.674 for 120 dB startle; Figure 1B).

### Tests for anxiety-like behavior

We found a significantly decreased locomotor activity under the stressful conditions in CS-KO mice in the open field test, the elevated plus maze, and the social interaction test. No significant changes were seen in any indices in the light/dark transition test. Detailed results are described as follows (supplemental data are available on-line).

#### Open field test (general locomotor activity and emotionality)

**Figure 2:**
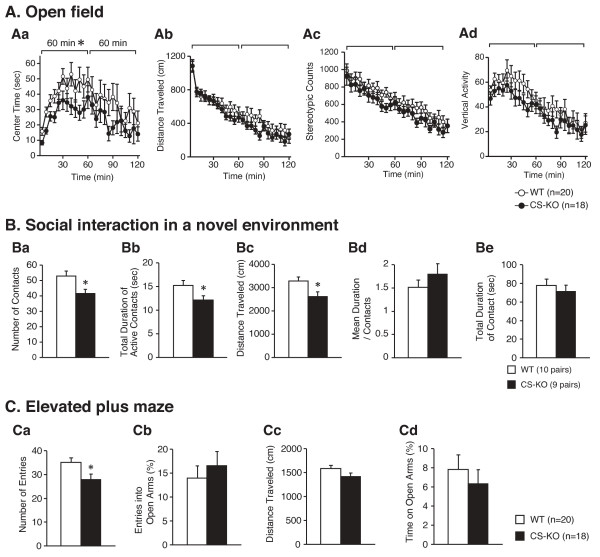
**Decreased activity of CS-KO mice in the tests for anxiety-like behavior**. In the open field test (A), time spent in center was not significantly different between genotypes during the whole 120 min period. However, in the first 60 min of the trial, CS-KO mice spent significantly less time in the center of the field (Aa). The total distance traveled was not significantly affected by the lack of CS (Ab). Stereotypic counts were not significantly different between genotypes (Ac). No significant difference was detected between genotypes in vertical activity (Ad). During a social interaction test in a novel environment (B), the number of contacts (Ba) and the total duration of active contacts (Bb) of CS-KO mice were significantly smaller than those of WT mice. However it is possible that this is due to decreased locomotor activity of the CS-KO mice in this test (Bc). The mean duration of contact (Bd) and total duration of contacts (Be) in CS-KO mice did not differ significantly from that of WT mice. In the elevated plus maze (C), CS-KO mice showed a significant decrease in number of entries (Ca), and tendency of decrease in distance traveled (Cc). Neither percentage entries into open arms (Cb) nor time on open arms (Cd) was affected by the lack of CS. (*) Significantly different during first 60 min block in genotype effect, *P *< 0.05. Data are expressed as mean ± S.E.M.

During the whole 120 min period of open field test, we did not detect significant differences between genotypes in time spent in center (F_1,36 _= 3.16, *P *= 0.0841, Figure 2Aa), total distance traveled (F_1,36 _= 1.12, *P *= 0.297, Figure 2Ab), stereotypic counts (F_1,36 _= 1.46, *P *= 0.234, Figure 2Ac), and vertical activity (F_1,36 _= 1.21, *P *= 0.278, Figure 2Ad). There was a tendency of decrease in activity in CS-KO mice, especially in the first 60 min period. In the first 60 min of the trial, CS-KO mice spent significantly less time in the center of the open field apparatus (F_1,36 _= 4.57, *P *= 0.0394 in first 60 min; F_1,36 _= 1.60, *P *= 0.215 in the following 60 min) (Figure 2Aa). The total distance traveled was not significantly affected by the lack of calpastatin (F_1,36 _= 0.383, *P *= 0.540 in the first 60 min; F_1,36 _= 1.56, *P *= 0.220 in the following 60 min) (Figure 2Ab). Stereotypic behavior counts (F_1,36 _= 1.25, *P *= 0.272 in the first 60 min; F_1,36 _= 1.25, *P *= 0.271 in the following 60 min) (Figure 2Ac), and vertical activity (F_1,36 _= 1.77, *P *= 0.192 in the first 60 min; F_1,36 _= 0.597, *P *= 0.445 in the following 60 min) (Figure 2Ad) were not significantly affected by the lack of CS.

#### Social interaction tests (sociability and anxiety-like behavior)

During a 10-min social interaction test in a novel environment, the number of contacts and the total duration of active contacts of CS-KO mice were significantly lower than those of WT mice (F_1,17 _= 7.05, *P *= 0.0166; Figure 2Ba and F_1,17 _= 4.92, *P *= 0.0405; Figure 2Bb, respectively). However, because the locomotor activity of the CS-KO mice was decreased in this test (F_1,17 _= 6.44, *P *= 0.0213; Figure 2Bc), it is possible that their decreased number of contacts and total duration of active contacts were consequences of the decreased locomotor activity. The mean duration of contact and total duration of contact in CS-KO mice did not differ significantly from those of WT mice (F_1,17 _= 1.15, *P *= 0.299; Figure 2Bd and F_1,17 _= 0.504, *P *= 0.487; Figure 2Be, respectively). We could not find noticeable differences between the genotypes in Crawley's Sociability and social novelty preference test [see Additional file 1] and home cage social interactions [see Additional file 2].

#### Elevated plus maze test (anxiety-like behavior)

CS-KO mice showed a significant decrease in the total number of entries into arms (F_1,36 _= 5.98, *P *= 0.0195; Figure 2Ca), and also showed a tendency of decrease in distance traveled (F_1,36 _= 3.30, *P *= 0.0776; Figure 2Cc). Neither percentage entries into open arms or time on open arms was affected by the lack of CS (F_1,36 _= 0.46, *P *= 0.832 in percentage entries into open arms; Figure 2Cb, F_1,36 _= 0.491, *P *= 0.488 in time on open arms; Figure 2Cd).

#### Light/dark transition test (anxiety-like behavior)

CS-KO mice showed no significant differences in the light/dark transition test in distance traveled [see Additional file 3] (F_1,36 _= 0.011, *P *= 0.917 in light camber, F_1,36 _= 0.293, *P *= 0.592 in the dark chamber; Aa), in stay time in the light chamber (F_1,36 _= 0.158, *P *= 0.694; Ab), in the number of transitions (F_1,36 _= 0.448, *P *= 0.508; Ac), or in latency to first entry into the light chamber (F_1,36 _= 0.006, *P *= 0.936; Ad).

### Tests for learning and memory

#### Eight-arm radial maze (working memory)

**Figure 3:**
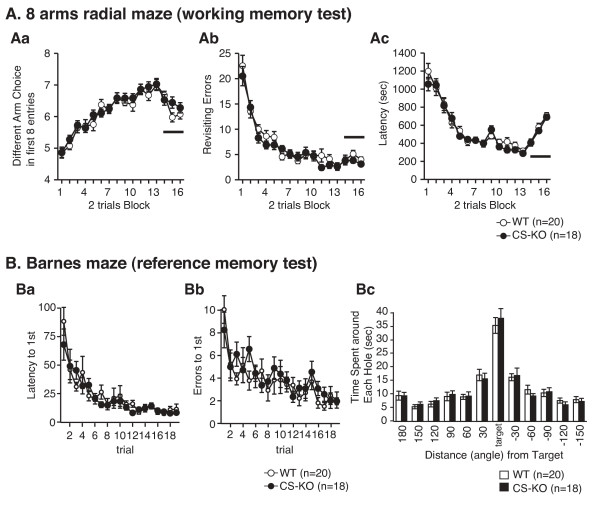
**Normal memory ability of CS-KO mice in eight-arm radial maze and Barnes maze**. In the eight-arm radial maze (A), we could not detect a significant difference between genotypes in the different arm choices in first 8 entries (Aa), in the number of revisiting errors (Ab), or in latency to take all the pellets (Ac). The bold horizontal bars in graph Aa-Ac indicate the delay task sessions (Delay duration: 30, 120, and 300 sec from the left respectively). In Barnes maze (B), we could not detect a significant difference between genotypes in either the latency to reach the correct hole above the escape box (Ba) or in the number of trials to the first entry into the escape box (Bb) of acquisition trials. In probe trial (transfer test) performance on day 10 (Bc), CS-KO mice appeared normal as regards the time spent around each escape hole. Data are expressed as mean ± S.E.M.

The number of different arm choices in the first 8 entries, the number of revisiting errors, and latency to obtain all the food reinforcers were not significantly different between genotypes (F_1,36 _= 0.719, *P *= 0.402 in different arm choice in the first 8 entries; Figure 3Aa, F_1,34 _= 0.847, *P *= 0.363 in revisiting errors; Figure 3Ab, F_1,36 _= 0.222, *P *= 0.640 in latency to take all the foods; Figure 3Ac). Applying the delay time toward the end of testing disturbed the test score in a delay duration time-dependent manner, but no significant difference between genotypes was detected.

#### Barnes maze acquisition (reference memory)

Barnes maze acquisition was not significantly different between the CS-KO mice and their WT controls. Acquisition trials revealed that the latency to reach the correct hole above the escape box was not significantly different in the CS-KO mice (F_1,34 _= 0.460, *P *= 0.502; Figure 3Ba). The number of errors in the first entry into the escape box was not significantly different in the CS-KO mice (F_1,34 _= 0.797, *P *= 0.378; Figure 3Bb). In the probe trial (transfer test) that was conducted one day after the last training, CS-KO mice appeared normal in terms of the time spent around each escape hole (F_1,34 _= 0.060, *P *= 0.808; Figure 3Bc).

#### Contextual and cued fear conditioning test (classical conditioning)

**Figure 4:**
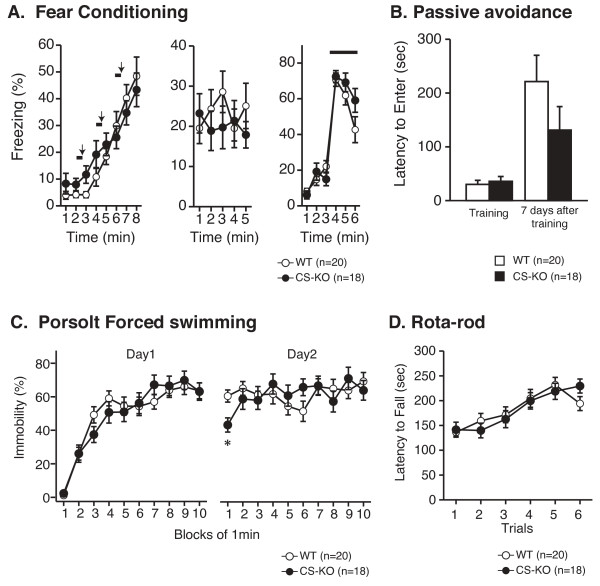
**Normal memory ability of CS-KO mice in the other memory-related tests (fear learning, depression learning, and motor learning)**. In the fear conditioning test (A), the freezing response with tone-shock conditioning was normal in CS-KO mice (left). Freezing during contextual testing was not significantly different between genotypes (center). In cued testing, CS-KO mice showed essentially normal freezing responses (right). During the time indicated by horizontal bold lines, tone stimuli were given. Arrows indicate the time points of foot shock stimuli. In the passive avoidance test (B), CS-KO mice showed normal latency to enter the dark side of the passive avoidance chamber, compared with WT mice in training (B left). Seven days after training, the latency to enter the dark side significantly increased compared with the training trial in both genotypes (B right). We detected a small, but not significant decrease in CS-KO mice in day 7. In the Porsolt forced swimming test (C), CS-KO mice displayed a normal immobility during the whole 10-min forced swimming session (C left). On the next day, during the whole 10-min session, immobility characteristics seemed to be the same in the two genotypes (C right). (*) CS-KO mice displayed decreased immobility compared with WT mice only in the first 1 min block (*P *= 0.0037). Rotarod performance of CS-KO mice (D) was normal compared with WT mice during the trials. Data are expressed as mean ± S.E.M.

In the contextual and cued fear conditioning test, freezing responses to a mild foot-shock stimulus and leading sound conditioning stimulus were induced normally in CS-KO mice (F_1,36 _= 0.227, *P *= 0.636; Figure 4A left). In the following trial (using the same chamber as used in the previous conditioning, without the sound cue), the learned freezing response of CS-KO mice was induced normally (F_1,36 _= 0.404, *P *= 0.529; Figure 4A center). In the cued trial (putting the mice into a different shape of chamber from that used for the conditioning, but with the presence of sound as used for the conditioning), CS-KO and WT mice displayed similar increased freezing responses to the tone (F_1,36 _= 0.742, *P *= 0.395; Figure 4A right).

#### Passive avoidance test (contextual memory)

In the training trial, CS-KO mice showed normal latency to enter the dark chamber of the passive avoidance apparatus in a manner comparable to WT mice (F_1,36 _= 0.242, *P *= 0.626). Seven days after the training, latency to enter the dark side significantly increased compared with day one (F_1,34 _= 15.0, P = 0.0004, in WT mice; F_1,34 _= 4.57, P = 0.0399, in CS-KO mice), with no significant difference of latency between genotypes (F_1,36 _= 1.86, *P *= 0.181) (Figure 4B).

#### Porsolt forced swimming test (depression-like behavior)

In the Porsolt forced swimming test, CS-KO mice displayed a normal immobility during the whole 10-min forced swimming session (F_1,36 _= 0.031, *P *= 0.861; Figure 4C left). On the next day, immobility was greater than that on the first day from the beginning of the session in both genotypes. During the whole 10-min session, immobility characteristics were not significantly different between the two genotypes (F_1,36 _= 0.031, *P *= 0.862; Figure 4C right). The interaction between genotype × time effect was not significant between genotypes on day 2 (F_9,36 _= 1.56, P = 0.127), but there was an apparent difference in immobility between genotypes with CS-KO mice displaying significantly decreased immobility compared with WT mice only in the first 1 min block (F_1,36 _= 7.73, *P *= 0.0086; Figure 4C right).

#### Rotarod test (associative motor learning)

Rotarod performance of CS-KO mice was normal compared with WT mice during the trials (F_1,36 _= 0.003, *P *= 0.960; Figure 4D).

## Discussion

To examine the physiological and pathophysiological roles of calpastatin, we have generated CS-KO mice [5], and conducted comprehensive behavioral analyses of these mice. No obvious differences were observed between CS-KO mice and littermate controls in the average life span, reproductive characteristics, or development [5]. We however found significant behavioral changes in CS-KO mice: decreased locomotor activity in stressful environments and decreased auditory startle response.

We found that in CS-KO mice, center time in the first 60 min of the open field test was significantly lower than that of WT. In the elevated plus maze, the number of entries into the arms was decreased in CS-KO mice. These results consistently indicate increased anxiety-like behavior in CS-KO mice in stressful environment. CS-KO mice also showed decreased social behavior in a novel environment. Since the social interaction test was originally developed to provide an ethologically based measure that was sensitive to both anxiolytic and anxiogenic drug effects, this test is also sensitive to a number of environmental and physiological factors that can affect anxiety [17]. We have detected increased anxiety-like behaviors in CS-KO mice (indicated by a decrease in distance traveled) using a social interaction test with a freely moving stranger mouse. However, in Crawley's sociability and social novelty test, which does not allow stranger mice to move freely, the exploration time around the stranger was not significantly different between CS-KO mice and WT mice. On the other hand, in another social interaction test in which a stranger mouse can move freely, we detected decreased social behavior in CS-KO mice. This apparent discrepancy between the results of two social interaction tests may be due to the mobility of stranger mice. Stranger mouse that moves freely may represent more stressful stimulus to the experiment mice. These data are consistent with decrease of activity in stressful environments in CS-KO mice. In a previous behavioral study, μ-calpain KO mice displayed more rearing behavior before conditioning in a fear-conditioning chamber compared with WT mice [12]. Rearing is not a common measure of anxiety-like behavior, but decreased anxiety-like behavior may allow increased exploration in a novel environment. The correspondence of behavioral experiment results between μ in symbol]-calpain KO mice and CS-KO mice also supports the possibility that the calpain-CS system regulates anxiety-like behavior. We detected increased anxiety-like behavior of CS-KO mice in the open field, elevated plus maze, social interaction and fear conditioning tests, but not in the light-dark transition test. How can we explain this apparent discrepancy? The comprehensive behavioral test battery in our report has been conducted with identical protocols on more than 70 different knockout and transgenic mice over the past five years [15]. By a factor analysis using large set of data on more than 5000 mice from this test battery, we are aware that these anxiety-like behavior tests assess different aspects of anxiety-like behavior, although there are significant correlations between indices in some of these tests [18]. There are also published data indicating that different anxiety tests measure different aspects of anxiety-like behaviors [19-22], demonstrating the fact that one but not the other assay could provide evidence of altered anxiety-like behavior. To distinguish different anxiety-related behavioral dimensions, CS-KO mice would have to be tested further by other anxiety-related behavioral tests.

We failed to detect any significant alterations of learning and memory performances in CS-KO mice. A previous study indicated an increased degree of synaptic potentiation in the Milan hypertensive strain of rat, which is known to have genetic deficiencies including decreased calpastatin expression [13]. LTP in the hippocampus is the leading experimental model for the synaptic changes that may underlie learning and memory [9], and it has been hypothesized that calpain is involved in the process of memory formation [6,7,23-25]. We also detected higher LTP after tetanus stimulation in CA1 and dentate gyrus of anesthetized CS-KO mice (Huang and Saido, unpublished data). Therefore, we applied learning and memory tests to CS-KO mice. We used the 8-arms radial maze to compare spatial working memory between genotypes. This test is thought to be prefrontal cortex- and hippocampus-dependent in rodents [26,27].

However, no significant differences between the genotypes were detected (trials 1–32 and delay trials). We also evaluated spatial reference memory using the Barnes maze [28]. As learning occurs, latency to first entry into the escape hole and the number of errors involving choosing incorrect holes decrease. In a probe test trial, we found no significant difference between genotypes in time around the correct hole where the escape box had been located. We could not detect any significant alterations in either working memory or reference memory in CS-KO mice in these tests. How can we explain the disconnection between LTP results and the lack of alteration in learning? There is a report showing enhanced hippcampal LTP but impaired learning in PSD-95 KO mice, and suggest that training of PSD-95-mutant mice in a spatial learning task might cause too many synapses within the network to become strongly potentiated and not enough to be depressed, resulting in a degradation of information storage and recall capacity, which manifests as a learning impairment [29]. Spectrin, the major target of calpain, is, like PSD-95, also suggested to be related to the localization of glutamate receptors [30], If calpain is hyperactivated in our mice, higher spectrin degradation may allow overexpression of glutamate receptors, but this does not have to be the reason of enhanced spatial memory. Furthermore, there is a paper clarifying that output from hippocampal CA3 to entorhinal cortex via CA1 (trisynaptic pathway) is dispensable and the monosynaptic pathway (entorhinal cortex – CA1 – entorhinal cortex) is sufficient for incremental spatial learning. From these two reports, we can say that increased LTP in the trisynaptic pathway does not necessarily affect incremental spatial learning [31]. Based on these two reports, it is unlikely that increased LTP in the trisynaptic pathway is essential for incremental spatial learning.

Contextual and cued fear conditioning test can be used to examine both hippocampus-dependent memory and amygdala-dependent emotional memory [32-34]. There were no significant genotype effects on fear memory in these tests. It has also been reported that μ-calpain KO mice showed normal freezing behavior in contextual and cued tone fear conditioning test [12]. In addition, in the passive avoidance test, we also failed to find significant changes in memory ability in this test. In every aspect, we failed to detect any significant alterations of memory ability in CS-KO mice. These observations argue against a major role of CS in learning and memory.

We have observed increased anxiety-like behavior in CS-KO mice, and these mice have higher LTP in hippocampal dentate gyrus region and CA1 region (Huang and Saido, unpublished data). Are these phenotypes connected to each other? N-methyl-D-aspartate (NMDA) receptor antagonists that are known to disrupt LTP in these regions have been reported to have anxiolytic effects in rodents [35,36]. According to these reports, higher NMDA receptor activation may cause increased anxiety-like behavior. Lynch and Baudry [37] have suggested that tetanic stimulation causes an increase in the postsynaptic Ca^2+ ^concentration which activates μ-calpain. Then μ-calpain cleaves spectrin, which allows glutamate receptors located deep in the postsynaptic membrane to the surface [38]. If NMDA receptor expression is regulated in this manner, it can be one of the possible explanations of increased anxiety-like behavior of CS-KO mice. Furthermore, because calpain is ubiquitous protease, there is a possibility that higher LTP is also observed in the amygdala, the region that regulates anxiety-like behavior. Further studies are needed to clarify the possible connection of higher LTP and higher anxiety-like behavior in CS-KO mice.

In contrast, we discovered an unexpected phenotype in CS-KO mice: the decrease of auditory startle response by 100 or 120 dB sound. This decrease of startle response may not be to due inner hair cell loss [39,40] nor muscle dysfunction [41], because we detected normal inhibition of the startle response by prepulse sounds in CS-KO mice, suggesting these mice could respond to the sounds. These results suggest a dysfunction within the auditory-startle response pathways, which consist essentially of an initial central relay in the cochlear nuclear complex, an intermediate brain stem relay in the reticular formation, a long reticulospinal pathway via the medial longitudinal fasciculus and outputs via spinal cord and brain stem motoneurons [42]. (Variance of calpain and CS expression level may be one possible explanation of this acoustic startle response deficiency. This will be discussed at the later part.)

The CS-KO mice were essentially normal in most of the behavior tests, indicating that CS plays a limited role in cognition. We did however observe significant abnormalities in affective responses. How can these behavioral abnormalities expressed by the lack of CS be accounted for? Takano et al. [5] demonstrated that calpastatin is a negative regulator of calpain under pathological conditions, implying that CS may not be fully responsible for inhibition of calpain activation and action under the normal conditions. On the other hand, a previous study showed increased calpain-dependent β-catenin cleavage in vivo upon transfer of mice into a novel and environmentally enriched cage [43], suggesting the possibility of change in calpain activity in response to stressor. Our CS-KO mice may show increased β-catenin cleavage in stressful conditions. Since we detected decreases of locomotor activity under stressful environments in CS-KO mice, CS may be one of the regulators that prevent calpain activation induced by environmental changes.

Difference of expression levels in molecules may also explain these behavioral phenotypes. The effects of CS deprivation can be higher in the regions which have higher calpain expression. Both μ-calpain and m-calpain are known as ubiquitous calpains [44], and are expressed to some degree in all brain regions. There are no detailed histology or western blotting reports comparing protein expression/activation levels between brain regions. Calpain can be activated very easily by experimental manipulations, which may be one reason for this difficulty. On the other hand, comparisons of mRNA expression levels are available at Allen Brain Atlas [see Additional file 4] [45-48]. Remarkably, mRNA expression densities are highest in medulla and pons, which are components of the central auditory pathways [49]. This expression is consistent with our acoustic startle response results. CS-KO mice may have some impairment in synaptic transmission of acoustic pathway in medulla and pons.

To clarify the actual expression mechanisms of these phenotypes, further experiments are needed. Our observations also suggest that the CS-mediated pathways are potential targets in therapeutic approaches to neurotic or psychotic symptoms. Although none of the commercially available calpain inhibitors are specific enough, membrane-permeable calpain inhibitors, if generated, could be candidate anti-anxiety medications.

## Conclusion

In conclusion, we have identified novel phenotypes of CS-KO mice: decreased locomotor activity in stressful environments. We also detected an impairment of the auditory startle response. Lack of CS did not affect either spatial memory or amygdala-dependent memory, which implies CS may not be necessary for learning and memory mechanisms. These findings provide clues to physiological roles of CS-regulated pathways.

## Methods

### Generation of CS-KO mice

Male CS-KO mice and their wild-type littermate controls (WT) were generated by mating heterozygous CS (+/-) mice as described elsewhere [5]. They were bred for 11 generations on C57BL/6J background. KO and WT for the experiments were obtained by crossing N11 CS (+/-) mice.

### Experimental Design

Behavioral tests were started when animals were 2.5 months old; by the end of the test battery, mice were 6.5 months of age. Mice were housed in a room with a 12-hr light/dark cycle (lights on at 7:00 a.m.) with access to food and water ad libitum. Animals were housed in groups consisting of two WT mice and two CS-KO mice per cage. Our behavioral test battery consists of general health and neurological screening, light/dark transition test, open field test, elevated plus-maze test, social interaction tests, wire hang, rotarod, hotplate, prepulse inhibition, Porsolt forced swimming test, Barnes maze test, 8-arm radial maze, cued and contextual fear conditioning, passive avoidance and 24-hour social interaction test in the home cage (listed in actual order of performance). Behavioral testing was performed between 9:00 a.m. and 6:00 p.m. except for the 8-arm radial maze test which was performed between 7:00 p.m. and 5:00 a.m. The experimental room was illuminated at 100 lux (measured at the top of the rack where mouse cages were placed for habituation), unless specified otherwise. The experimental chamber was illuminated at 100 lux in the open filed test without room illumination, and for the Barnes maze, the experimental room was illuminated at maximum intensity (800 to 1000 lux). Before performing the tests, all apparatus were cleaned with "Super hypochlorous water" (Shimizu Laboratory Supplies co., ltd., Kyoto, Japan) to minimize the effect of olfactory stimulus on behaviors. All procedures were performed according to the guidelines of the Animal Use and Care Committees of RIKEN and Kyoto University.

### Video-tracking system

During the behavioral experiments, movies of mice were taken by CCD camera and stored as sequential TIFF files. These files were analyzed automatically by software based on a public domain program, NIH Image or Image J [50] Applications were specifically designed for each task as described below. The correlation of the judge for freezing between human observation and image analysis was greater than 0.95 (data not shown) during the Porsolt forced swim test, the tail suspension test and the fear conditioning test. The correlation of the measurement of distance traveled between the Accuscan system and the video tracking system was greater than 0.9. Those results support the validation of our Image analysis software for behavioral phenotyping.

### Neurological screening

Physical features, including the presence of whiskers or bald patches, were recorded. Righting reflex, response to a whisker touch from behind (whisker twitch), response to a ear touch from behind (ear twitch reflexes), and reflex by key-jangling sound were evaluated.

### Startle response/prepulse inhibition tests

A startle reflex measurement system was used (Ohara & Co., Tokyo). A test session was started by placing a mouse in a Plexiglas cylinder, where it was left undisturbed for 10 min. The duration of white noise that was used as the startle stimulus was 40 msec for all trial types. The startle response was recorded by accelerometer for 140 msec (measuring the response every 1 msec) starting with the onset of the prepulse stimulus. The background noise level in each chamber was 70 dB. The peak startle amplitude recorded during the 140 msec sampling window was used as the dependent variable. A test session consisted of 6 trial types (i.e. two types for startle stimulus-only trials, and four types for prepulse inhibition trials). The intensity of the startle stimulus was 110 or 120 dB. The prepulse sound was presented 100 msec before the startle stimulus, and its intensity was 74 or 78 dB. Four combinations of prepulse and startle stimuli were employed (74–110, 78–110, 74–120, and 78–120). Six blocks of the 6 trial types were presented in pseudorandom order such that each trial type was presented once within a block. The average inter-trial interval was 15 sec (range: 10–20 sec).

### Hot plate test

A hot plate test was used to evaluate the sensitivity to a painful stimulus. Mice were placed on a hot plate (Columbus Instruments, Columbus, Ohio) at 55.0 (± 0.3)°C, and latency to the first paw response was recorded. The paw response was either a foot shake, or a paw lick, or lifting both forepaws simultaneously.

### Wire hang test

A wire hang test apparatus (Ohara & Co., Tokyo) was used to assess balance and grip strength. The apparatus consists of a box (21.5 × 22 × 23 cm) with a wire mesh grid (10 × 10 cm) on its top, which can be inverted. The mouse was placed on the wire mesh, which was then inverted, causing the animal to grip the wire. Latency to fall was recorded, with a 60 sec cut-off time.

### Grip strength test

A grip strength meter (Ohara & Co., Tokyo) was used to assess forelimb grip strength. Mice were lifted and held by their tail so that their forepaws could grasp a wire grid. The mice were then gently pulled backward by the tail with their posture parallel to the surface of the table until they release the grid. The peak force applied by the forelimbs of the mouse was recorded in newtons (N). Each mouse was tested three times and the highest value obtained was used for statistical analysis.

### Open field test

Locomotor activity was measured using an open field test. Each subject was placed in the center of the open field apparatus (40 × 40 × 30 cm; Accuscan Instruments, Columbus, Ohio) equipped with photocells (beam spacing 2.5 cm, beam diameter 4 mm, beam frequency 50 times/s). Total distance traveled (in cm), vertical activity (rearing measured by counting the number of beam interruptions), time spent in the center area of the open field and stereotypic counts were recorded by the VersaMax system. Center area was defined as 1 cm away from the walls. If the beam at the edge of the open filed, that was 1 cm apart from the wall, was not interrupted, mice were considered to be in the center area. If mice broke the same beam (or set of beams) repeatedly then they were considered to be exhibiting stereotypic activity. This typically happens during grooming, head bobbing, etc. Stereotypic counts are the number of beam breaks that occur during the period of stereotypic activity. Data were collected for 120 min.

### Social interaction test in a novel environment (single chamber)

In the social interaction test, two mice of identical genotype, which had previously been housed in different cages, were placed in a box together (40 × 40 × 30 cm) and allowed to explore freely for 10 min. Social behavior was monitored with a CCD camera connected to a Macintosh computer. Analysis was performed automatically, using Image J based original program (Image SI: see Data analysis). The number of contacts, mean duration per contact (sec) and total distance traveled were measured.

### Eight-arm radial maze test

The eight-arm radial maze test was conducted in a manner similar to that described previously [51]. The floor of the maze was made of white plexiglas, and the wall (25 cm high) consisted of transparent plexiglas. Each arm (9 × 40 cm) radiated from an octagonal central starting platform (perimeter 12 × 8 cm) like the spokes of a wheel. Identical food wells (1.4 cm deep and 1.4 cm in diameter) with pellet sensors were placed at the distal end of each arm. The pellet sensors were able to automatically record pellet intake by the mice. The maze was elevated 75 cm above the floor and placed in a dimly lit room with several extra-maze cues. During the experiment, the maze was maintained in a constant orientation. One week before pretraining, animals were deprived of food until their body weight was reduced to 80–85% of the initial level. Pretraining started on the 8th day. Each mouse was placed in the central starting platform and allowed to explore and to consume food pellets scattered on the whole maze for a 30 min period (one session per mouse). After completion of the initial pretraining, mice received another pretraining to take a pellet from each food well after being placed at the distal end of each arm. A trial was finished after the subject consumed the pellets. This pretraining was repeated 8 times, using the 8 different arms, for each mouse. After these pretraining trials, actual maze acquisition trials were carried out. All 8 arms were baited with food pellets. Mice were placed on the central platform and allowed to get all 8 pellets within 25 min. A trial was terminated immediately after all 8 pellets were consumed or after 25 min had elapsed. An 'arm visit' was defined as traveling for more than 5 cm from the central platform. The mice were confined in the central platform for 5 sec after each arm choice. The animals went through one trial per day (32 trials total). For each trial, choices of arms, latency to get all pellets, distance traveled, number of different arms chosen within the first 8 choices, and the numbers of revisiting and omission errors were automatically recorded. Data acquisition, control of guillotine doors, and data analysis were performed using Image J based original program (Image RM: see Data analysis). During the 27th and 28th (= 14th block) acquisition trial, a 30 sec delay was initiated after four pellets had been taken by confining the mice in the central platform. From the 29th and 30th trial (= 15th block), the delay period was extended to 120 sec and from the 31 and 32nd trial (= 16th block), 300 sec.

### The Barnes spatial navigation task

The Barnes task was conducted on "dry land," a white circular surface, 1.0 m in diameter, with 12 holes equally spaced around the perimeter (Ohara & Co., Tokyo). The circular open field was elevated 75 cm from the floor. A black Plexiglas escape box (17 × 13 × 7 cm), which had paper cage bedding on its bottom, was located under one of the holes. The hole above the escape box represented the target, analogous to the hidden platform in the Morris task. The location of the target was consistent for a given mouse, but was randomized across mice. The maze was rotated daily, with the spatial location of the target unchanged with respect to the visual room cues, to prevent a bias based on olfactory or proximal cues within the maze. Three trials per day were conducted for 9 successive days in the beginning (on days 5 and 6, no trial was undertaken). One day after the last training, a probe trial was conducted without the escape box, to confirm that this spatial task was acquired based on navigation using distal environment room cues. Latency to reach the target hole, distance to reach the target hole, number of errors, and time spent around each hole were recorded by video tracking software (Image BM).

### Contextual and cued fear conditioning

Each mouse was placed in a test chamber (26 × 34 × 29 cm) inside a sound-attenuated chamber (Ohara & Co., Tokyo) and allowed to explore freely for 2 min. A 60 dB white noise, which served as the conditioned stimulus, was presented for 30 sec, followed by a mild (2 sec, 0.5 mA) footshock, which served as the unconditioned stimulus. Two more of tone-shock stimulus pairings with same durations as the first stimulus were presented with a 2 min inter-stimulus interval (exposed at 2, 4, and 6 min time points). To examine shock sensitivity, we measured the distance traveled when the foot shock was delivered (from 2 sec before the shock to 2 sec after the shock, total 6 sec). Context testing was conducted one day after conditioning in the same chamber for 180 sec to each mouse. Cued testing with an altered context was conducted one day after conditioning using a triangular box (35 × 35 × 40 cm) made of white opaque Plexiglas, which was located in a different room. Tone stimulus for the cued testing was applied for 180 sec. Data acquisition, control of stimuli (i.e. tones and shocks), and data analysis were performed automatically, using using Image J based original program (Image FZ: see Data analysis) Images were captured at 1 frame per second. For each pair of successive frames, the amount of area (pixels) by which the mouse moved was measured. When this area was below a certain threshold (i.e., 20 pixels), the behavior was judged as 'freezing'. When the area equaled or exceeded the threshold, the behavior was considered as 'non-freezing'. The optimal threshold (number of pixels) to judge freezing was determined by adjusting it to the amount of freezing assessed by human observation. 'Freezing' that lasted less than the defined time threshold (i.e. 2 sec) was not included in the analysis.

### Passive avoidance

Passive avoidance (PA) apparatus was a trapezoidal light-dark box, consisting of a light chamber (10 × 14 cm at the top and 4.3 × 13 cm at the bottom) and a dark chamber (10 × 16 cm at the top and 4.3 × 16 cm at the bottom). The floor of both chambers was made of 1.2 mm stainless steel rods spaced 6 mm apart, the floor of the dark chamber could be electrified. The chambers were connected by guillotine door. The training trial was started by placing the animal inside the light chamber and raising the guillotine door. The animal spontaneously entered the dark chamber, receiving a footshock (0.3 mA) and returning to the light chamber. Seven days after this training trial, the latency to enter the dark chamber was measured. No exit from the non-shocked light chamber during the 300 s test period was considered successful passive avoidance.

### Elevated plus maze test

The elevated plus maze apparatus consisted of two open arms (25 × 5 cm) and two enclosed arms of the same size, with 15 cm high transparent walls. The arms and central square were made of white plastic plates and were elevated 55 cm above the floor. In order to minimize the likelihood of animals falling from the apparatus, 3-mm high plexiglas ledges were provided for the open arms. Arms of the same type were arranged at opposite sides to each other. Each mouse was placed in the central square of the maze (5 × 5 cm), facing one of the closed arms. Behavior was recorded during a 10 min test period. The number of entries into, and the time spent on, open and enclosed arms were recorded. For data analysis, we employed the following four measures: the percentage of entries into open arms, the stay time on open arms (sec), the number of total entries, and total distance traveled (cm). To specify the locations of the mice, the center of balance was used (i.e. "entry" means the entrance of center of balance to the other arm). Data acquisition and analysis were performed automatically, using using Image J based original program (Image EP: see Data analysis)

### Light/dark transition test

The apparatus used for the light/dark transition test consisted of a cage (21 × 42 × 25 cm) divided into two sections of equal size by a partition with a door (Ohara & Co., Tokyo). One chamber was brightly illuminated (390 lux), whereas the other chamber was dark (2 lux). Mice were placed into the dark area and allowed to move freely between the two chambers through the open door for 10 min. The total number of transitions and time spent on each side were recorded using using Image J based original program (Image LD4: see Data analysis) Full entries to the other chamber were regarded as one entry. Online material describing this method visually is available in the Journal of Visualized Experiment [52].

### Social interaction test in home cage

Social interaction monitoring in the home cage was conducted as previously described [53]. The system contains a home cage (29 × 18 × 12 cm) and a filtered cage top, separated by a 13-cm-high metal stand containing an infrared video camera, fitted on top of the stand. Two mice of the same inbred strain that had been housed separately were placed together in a home cage. Their social behavior was then monitored for a week. Outputs from the video cameras were fed into a Macintosh computer. Images from each cage were captured at a rate of one frame per second. Social interaction was measured by counting the number of particles in each frame: two particles indicated the mice were not in contact with each other; and one particle indicated contact between the two mice. We also measured locomotor activity during these experiments by quantifying the number of pixels that changed between each pair of successive frames. Analysis was performed automatically using Image SI software (see 'Image analysis').

### Crawley's sociability and social novelty preference test

Crawley's sociability and social novelty preference test is well-designed method to investigate the complex genetics of social behaviors [54]. The social testing apparatus consisted of a rectangular, three-chambered box and a lid with an infrared video camera (Ohara & Co., Tokyo). Each chamber was 20 × 40 × 22 cm and the dividing walls were made from clear Plexiglas, with small square openings (5 × 3 cm) allowing access into each chamber. An unfamiliar C57BL/6J male (stranger 1), that had had no prior contact with the subject mice, was placed in one of the side chambers. The location of stranger 1 in the left vs. right side chamber was systematically alternated between trials. The stranger mouse was enclosed in a small, round wire cage, which allowed nose contact between the bars, but prevented fighting. The cage was 11 cm in height, with a bottom diameter of 9 cm, vertical bars 0.5 cm and horizontal bars spaced 1 cm apart. The subject mouse was first placed in the middle chamber and allowed to explore the entire social test box for a 10-min session. The amount of time spent in each chamber was measured with the aid of a camera fitted on top of the box. Each mouse was tested in a 10-min session to quantify social preference for the first stranger. After the first 10-min session, a second unfamiliar mouse was placed in the chamber that had been empty during the first 10-min session. This second stranger was also enclosed in an identical small wire cage. The test mouse thus had a choice between the first, already-investigated unfamiliar mouse (stranger 1), and the novel unfamiliar mouse (stranger 2). The amount of time spent in each chamber during the second 10-minutes was measured as described above. Data acquisition and analysis were performed automatically, using Image J based original program (Image CSI: see Data analysis) software.

### Porsolt forced swimming test

The apparatus consisted of four Plexiglas cylinders (20 cm height × 10 cm diameter). The cylinders were filled with water (23°C), up to a height of 7.5 cm. Mice were placed in the cylinders, and the immobility and the distance traveled were recorded over a 10-min test period. Images were captured at one frame per second. For each pair of successive frames, the amount of area (pixels) within which the mouse moved was measured. When the amount of are was below a certain threshold, mouse behavior was judged as "immobile." When the amount of area equaled or exceeded the threshold, the mouse was considered as "moving." The optiminal threshold by which to judge was determined by adjusting it to the amount of immobility measured by human observation. Immobility lasting for less than a 2 sec. was not included in the analysis. Data acquisition and analysis were performed automatically, using using Image J based original program (Image TS: see Data analysis) software (see Data Analysis).

### Rotarod test

Motor coordination and balance were tested with the rotarod test, using an accelerating rotarod (UGO Basile Accelerating Rotarod). The mouse was placed on a rotating drum (3 cm diameter) and the time the animal was able to maintain its balance was measured. The speed of the rotarod was accelerated from 4 to 40 rpm over a 5-min period.

### Data analysis

Behavioral data were obtained automatically by applications that were based on the public domain NIH Image program and the Image J program and that were modified for each test by Tsuyoshi Miyakawa (available through OHara & Co., Tokyo, Japan). Statistical analysis was conducted using StatView (SAS Institute). Data were analyzed by two-way analysis of variance (ANOVA) or two-way repeated-measures ANOVA. Unless otherwise noted, the F and P values are for the genotype effect. The criterion for significance was set at *P *< 0.05.

## Competing interests

The authors declare that they have no competing interests.

## Authors' contributions

RN carried out breeding of experimental animals, all the data acquisitions (except Barnes maze and Fear conditioning), statistical analyses, experimental designs, and interpretations of behavioral data. KT carried out the data acquisitions of Barnes maze and fear conditioning, interpretations of behavioral data, experimental designing, and total coordination of all the experiments. SMH partly participated in the supervision of the work, proposed our working hypothesis and discussed the behavioral data. JT generated CS-KO mice and discussed the behavioral data. NI participated in supervision of the work and discussed the results. TM developed the comprehensive behavioral experiment systems including data acquisition software and method for analysis. TM supervised the behavioral experiments and discussed the results. TCS supervised all the aspects of the present study and discussed the results. All the authors helped to draft the manuscript.

## Supplementary Material

Additional file 1 Crawley's Sociability and social novelty preference test. We could not detect any significant difference between genotypes in exploratory behavior within either the first trial with one stranger mouse (F_1,34 _= 1.496, *P *= 0.2297 in the empty side; F_1,34 _= 0.242, *P *= 0.6256 in the stranger side; A), nor the following trial with an additional stranger mouse (F_1,34 _= 0.797, *P *= 0.3781 in the stranger 1 side; F_1,34 _= 0.105, *P *= 0.7483 in the stranger 2 side; B). The colors of mice in A and B, and the colors of lines under the bar graphs are corresponding to each other (white: empty side, light gray: stranger 1 side, dark gray: stranger 2 side). Click here for file

Additional file 2 Social interaction in home cage. Social interactions in home cage were normal in clustering (F_1,17 _= 0.54, *P *= 0.820; A). Activity level was not significantly different between genotypes either (F_1,17 _= 0.38, *P *= 0.547; B). Click here for file

Additional file 3 Light and dark transition test. CS-KO mice showed no significant differences in the light/dark transition test in distance traveled (F_1,36 _= 0.011, *P *= 0.917 in light camber, F_1,36 _= 0.293, *P *= 0.592 in the dark chamber; Aa), in stay time in the light chamber (F_1,36 _= 0.158, *P *= 0.694; Ab), in the number of transitions (F_1,36 _= 0.448, *P *= 0.508; Ac), or in latency to first entry into the light chamber (F_1,36 _= 0.006, *P *= 0.936; Figure Ad). Click here for file

Additional file 4 Comparison of mRNA expression levels of μ-calpain, m-calpain and calpastatin. Using Allen Brain Atlas, an in situ hybridization database of mouse brain, mRNA expression levels were compared among μ-calpain, m-calpain and calpastatin (Arranged to a bar chart by authors). Allen Brain Atlas [Internet]. Seattle (WA): Allen Institute for Brain Science. ^© ^2008. Available from: . Click here for file
