# Supplementary figures and images for: Comprehensive behavioral phenotyping of calpastatin-knockout mice
Source: Mol Brain. 2008 Sep 15;1:7. doi: 10.1186/1756-6606-1-7 (PMC2561015; doi:10.1186/1756-6606-1-7)

**A**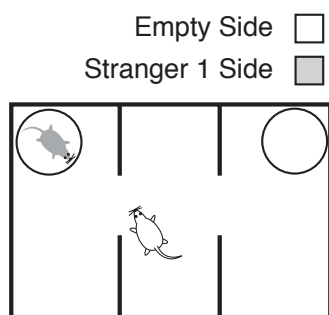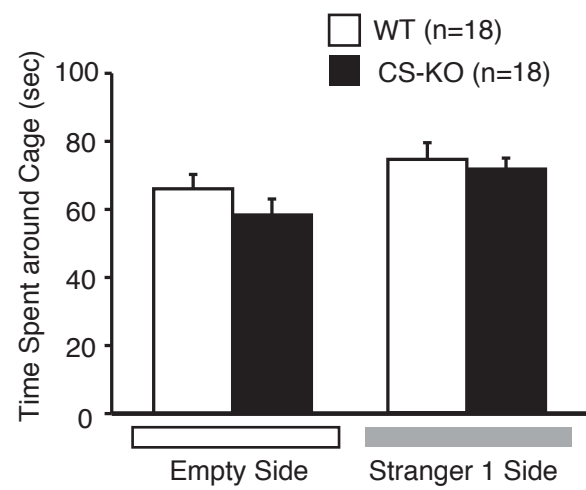**B**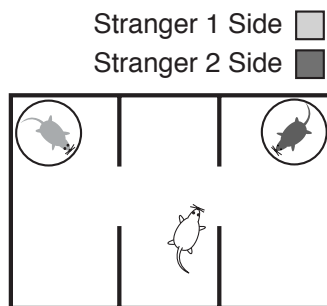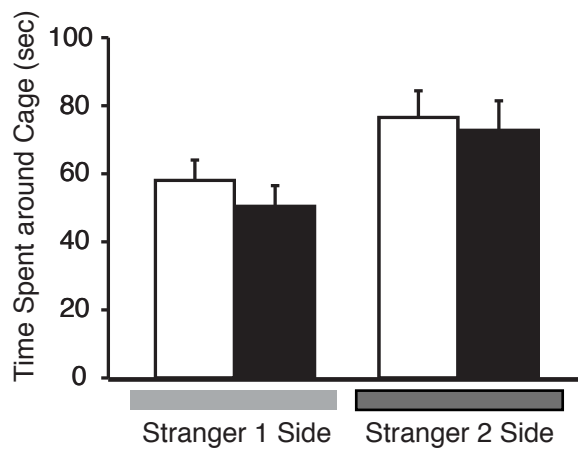

Supplement: Additional file 1 — Crawley's Sociability and social novelty preference test. We could not detect any significant difference between genotypes in exploratory behavior within either the first trial with one stranger mouse (F1,34 = 1.496, P = 0.2297 in the empty side; F1,34 = 0.242, P = 0.6256 in the stranger side; A), nor the following trial with an additional stranger mouse (F1,34 = 0.797, P = 0.3781 in the stranger 1 side; F1,34 = 0.105, P = 0.7483 in the stranger 2 side; B). The colors of mice in A and B, and the colors of lines under the bar graphs are corresponding to each other (white: empty side, light gray: stranger 1 side, dark gray: stranger 2 side). [file 1756-6606-1-7-S1.pdf]

**A**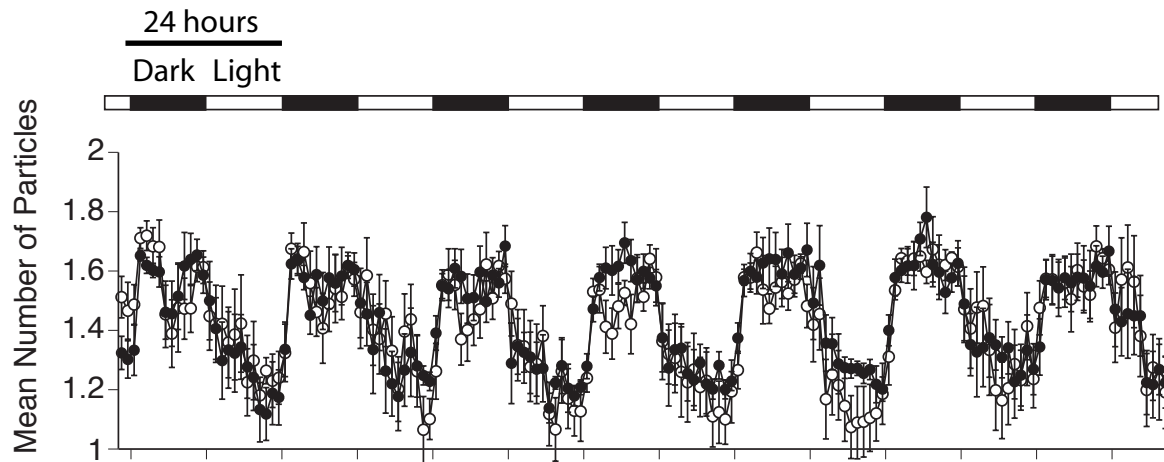**B**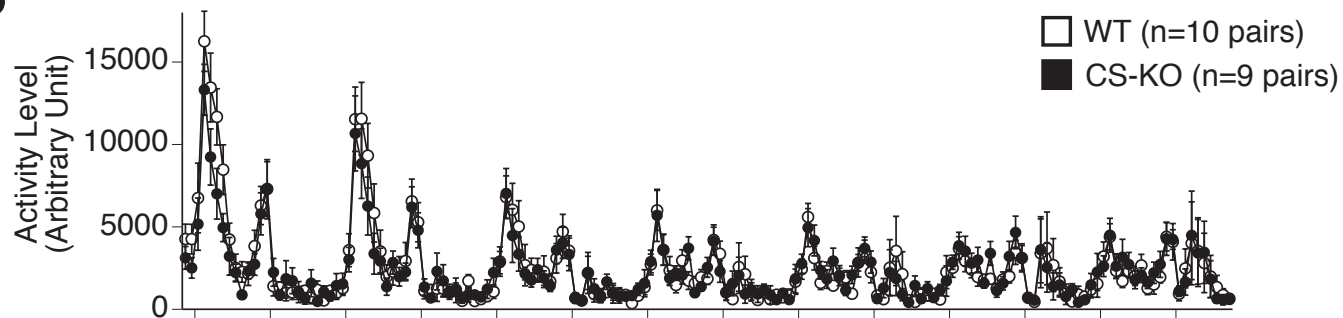

Supplement: Additional file 2 — Social interaction in home cage. Social interactions in home cage were normal in clustering (F1,17 = 0.54, P = 0.820; A). Activity level was not significantly different between genotypes either (F1,17 = 0.38, P = 0.547; B). [file 1756-6606-1-7-S2.pdf]

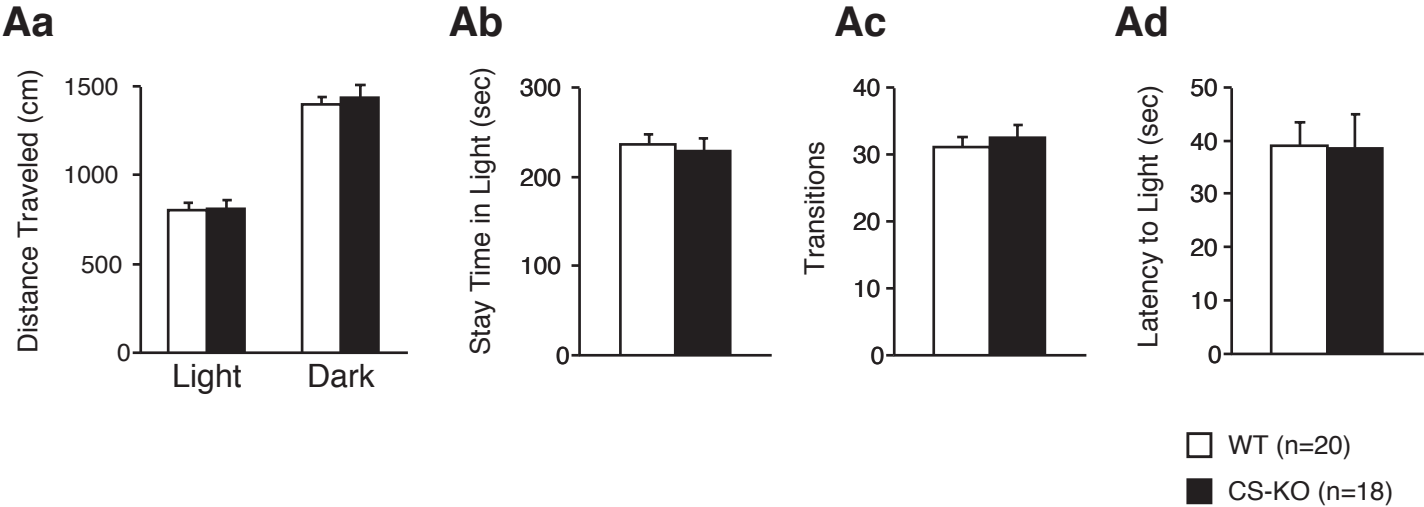

Supplement: Additional file 3 — Light and dark transition test. CS-KO mice showed no significant differences in the light/dark transition test in distance traveled (F1,36 = 0.011, P = 0.917 in light camber, F1,36 = 0.293, P = 0.592 in the dark chamber; Aa), in stay time in the light chamber (F1,36 = 0.158, P = 0.694; Ab), in the number of transitions (F1,36 = 0.448, P = 0.508; Ac), or in latency to first entry into the light chamber (F1,36 = 0.006, P = 0.936; Figure Ad). [file 1756-6606-1-7-S3.pdf]

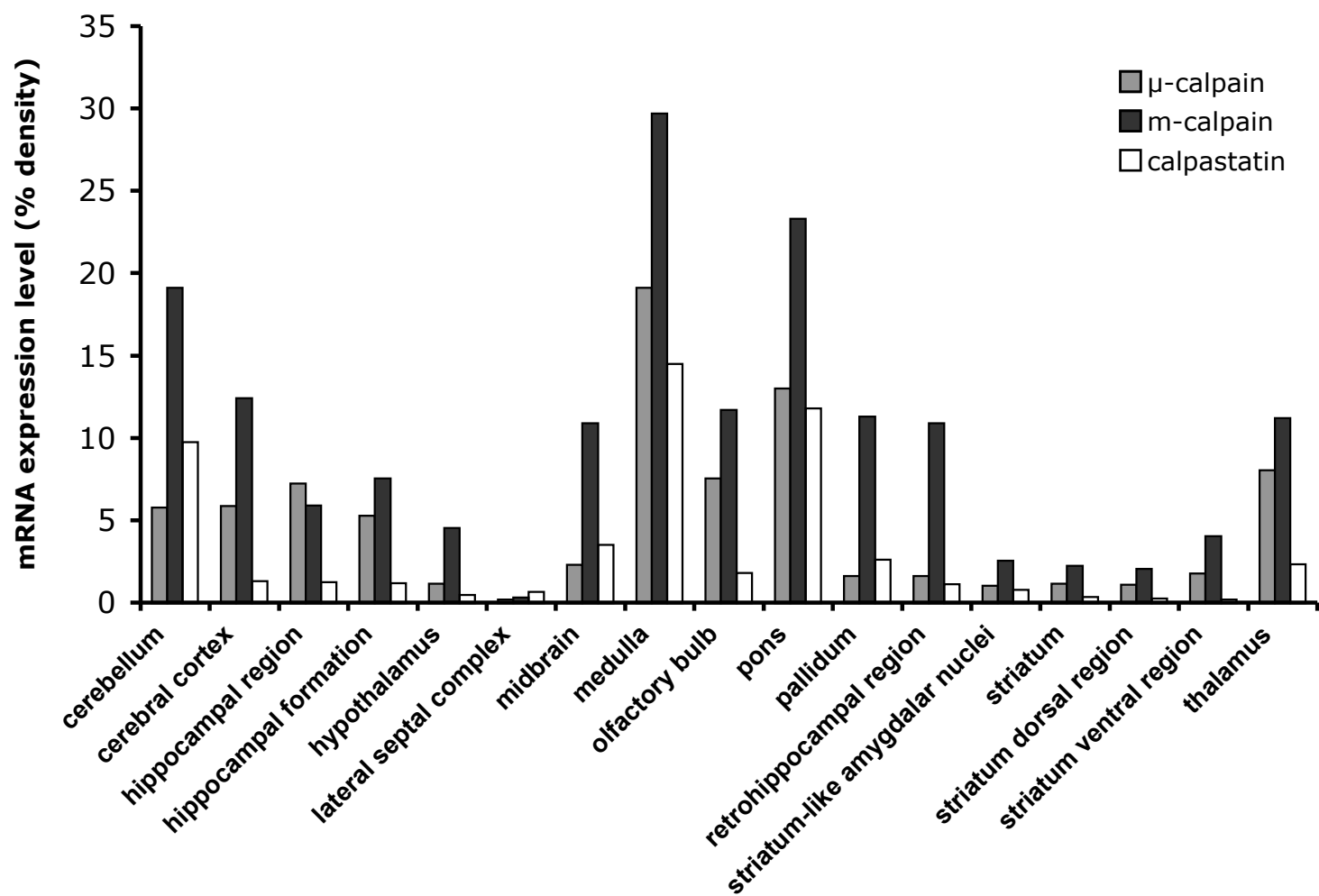

Supplement: Additional file 4 — Comparison of mRNA expression levels of μ-calpain, m-calpain and calpastatin. Using Allen Brain Atlas, an in situ hybridization database of mouse brain, mRNA expression levels were compared among μ-calpain, m-calpain and calpastatin (Arranged to a bar chart by authors). Allen Brain Atlas [Internet]. Seattle (WA): Allen Institute for Brain Science. © 2008. Available from: . [file 1756-6606-1-7-S4.pdf]
